# Demineralized bone matrix used for direct pulp capping in rats

**DOI:** 10.1371/journal.pone.0172693

**Published:** 2017-03-02

**Authors:** Qian Liu, Yanhong Ma, Junlan Wang, Xuefang Zhu, Yanjing Yang, Yufeng Mei

**Affiliations:** 1 Key Laboratory of Oral Diseases of Jiangsu Province and Stomatological School of Nanjing Medical University, Nanjing, Jiangsu, China; 2 Department of pediatric and preventive dentistry, The Affiliated Stomatological Hospital of Nanjing Medical University, Nanjing, Jiangsu, China; 3 Department of stomatology, Xuzhou Central Hospital, Xuzhou, Jiangsu, China; 4 The Affiliated Stomatological Hospital of Nanchang University, The Key Laboratory of Oral Biomedicine, Nanchang, Jiangxi, China; Texas A&M University Baylor College of Dentistry, UNITED STATES

## Abstract

**Objectives:**

To evaluate the wound healing process following direct pulp capping with demineralized bone matrix (DBM) and calcium hydroxide (Ca(OH)_2_).

**Methods:**

Fifty 8-weeks-old SPF Wistar male rats were divided into two groups: one was the DBM treated group, and the other was the Ca(OH)_2_ treated group. Pulpotomy was performed on the maxillary first molar of one side of each rat, and the another side was left as the blank control. Rats were sacrificed after each observation period (1, 3, 7, 14 and 28 days) and specimen slices were made. Hematoxylin-Eosin (HE) staining was used for observing the changes of pulp tissue, and immunohistochemical staining was used for observing the expression of reparative dentinogenesis-related factors runt transcription factor 2 (Runx2), type I collagen (COL I), osteocalcin (OCN) and dentin sialoprotein (DSP).

**Results:**

Inflammatory cell infiltration (ICI) and pulp tissue disorganization (PTD) could be observed in both the DBM and Ca(OH)_2_ groups at all observation periods. The DBM group showed slighter ICI on 1 and 28 days and milder PTD on 28 days, with a significant difference (P<0.05). Reparative dentin formation (RDF) could initially be observed on 14 days postoperatively, and the DBM group showed more regular and thinner RDF with significant differences on 14 and 28 days compared with the Ca(OH)_2_ group (P<0.05). In both groups, the expression of Runx2, COL I, DSP and OCN were positive. Generally, the expression of these four factors in the DBM group was stronger than the Ca(OH)_2_ group on the same observation periods.

**Conclusions:**

DBM had the ability of inducing odontoblast differentiation and promoting dentinogenesis. DBM could initiate physiologic wound healing in pulp and had the ability to promote reparative dentin formation. Consequently, DBM may be an acceptable alternative for direct pulp capping.

## Introduction

Dental pulp vitality is of great importance to teeth, not only for providing nutrition but also as biological sensors to detect outside stimulus. Preserving pulp vitality should always be the essential aim of endodontic treatment. Direct pulp capping as a viable and valid method to isolate outside stimulation has extraordinary significance [1, 2]. An ideal pulp capping material should isolate the pulp from infection and provide a biological environment for dental pulp tissue repair [3]. Calcium hydroxide (Ca(OH)_2_) pulp capping material has been used as the golden standard for a long time. However, Ca(OH)_2_ has its own limitations, such as inducing coagulation necrosis and pathologic calcification, pulp chamber obliteration [4, 5]. The new popular material, Trioxide aggregate (MTA), may efficiently induce reparative dentin formation (RDF) without inflammatory responses in the pulp. However, it is difficult to handle, exhibits poor adhesion to the tooth substrate, and has a latent impact on tooth color, which prevent its clinical use [6–8]. Thus, we attempt to seek a new direct pulp capping material that can meet the needs of promoting pulp repair with the slightest side effects.

DBM, which derives from natural bone tissue, is a biocompatible material and has been used in bone defect treatment [9, 10]. Its three-dimensional structure can provide cells anchorage sites, mechanical stability and structural guidance, which may result in new blood vessel invasion after been used localized [11]. DBM is mainly comprised of type I collagen(COL I) and bone morphogenetic proteins (BMPs) [12]. These two main components are favorable for the formation of dentin [13, 14]. COL I, as a component of dentine, can offer a scaffold for dental pulp cell migration as well as the attachment and deposition of the new dentin [13, 15]. COL I presents three-dimensional (3D)-porous structure in DBM. Among the 3D architecture, tissue cells interact with each other, and the extra cellular matrix results in a 3D communication network to maintain tissue homeostasis [16]. BMPs have been confirmed to promote the dental pulp stem cell differentiation into odontoblasts [17], which is the foundation of reparative dentine. BMPs not only regulate the development of tooth embryonic stem cells and odontoblasts differentiation but also participate in the dentin matrix secretion and mineralization [14, 18]. However, although COL I and BMPs both contribute to form dentine, no data have indicated that DBM can be used for direct pulp capping. Our study was proposed to compare the effects of direct pulp capping between DBM and Ca(OH)_2_. The results showed that DBM induced less inflammatory cell infiltration (ICI) and pulp tissue disorganization (PTD) suggesting the possibility of DBM used as a new pulp capping agent.

## Materials and methods

DBM was provided by Zhenghai Biotechnology Inc. (Yantai, Shandong, China). DBM has a 3D-porous structure with high porosity[19]. The small pieces of DBM (1–2 mm, diameter) looked like white sponge with pore sizes ranged from 350 to 750 μm and was sterilized packed.

### Experimental animals and experimental groups

Fifty eight-week-old male SPF Wistar rats (SLRC Laboratory Co., LTD, Shanghai, China) were randomly divided into two groups: the DBM group (n = 25) and the Ca(OH)_2_ group (n = 25). Unilateral maxillary first molar was used for the experimental group, the other side was left as the blank control group. The pulpotomy rat model was established as previously reported [20]. Before experimental operation, it took one week for all the rats to adapt the environment. All animal experiments were conducted in accordance with the accepted standards of animal care and approved by the Animal Ethical and Welfare Committee of Nanjing Medical University.

### Pulp-capping procedure

The rats were anesthetized with an intraperitoneal injection of 5% pentobarbital (Sigma, P3761, USA) at a dose of 40 mg/kg. After each rat fixed on an operating board, the mouth was kept open with a jaw prop. The teeth were disinfected with 2% iodine tincture and 75% alcohol cotton balls. A portable desktop electric dental handpiece (3000 rpm, Sae Yang Machinery Co., Taegu, Korea) with 1/4 drill long neck round bur (MANI. INC, Japan) were used on the mesial marginal ridge to open the pulp, and a 1/2 drill long neck round bur (MANI. INC, Japan) was used to remove the roof. A small spoon was used to remove the coronal pulp. During the procedure, the molars were constantly washed with 0.9% physiological saline. Hemostasis of the exposed pulp was achieved with 0.9% physiological saline cotton balls. After gentle air drying, the cavity was capped with DBM or Ca(OH)_2_ (Dycal, Dentsply International Inc., York, PA, USA), and glass ionomer cement (Fuji IX, GC, Tokyo, Japan) was placed on top of the cap as the rebase material. Finally, all the cavities were restored with resin (Filtek Z350 XT, 3M ESPE, St. Paul, MN, USA) with a light-curing unit for 20 s. After the operation, all rats were fed with a soft diet.

### Specimen preparation

Five observation periods were set: 1, 3, 7, 14, and 28 days. After each observation period, the rats were sacrificed by transcardial vital perfusion with 4% paraformaldehyde phosphate buffer solution (PFA, pH 7.4, Ling Feng chemical reagent co., LTD, Shanghai, China) under general anesthesia. The maxillae, along with the experimental teeth, were removed and immersed in 4% PFA at 4°C for another 3 hours for further fixation.

### Tissue preparation and serial sectioning

After fixation, the specimens were decalcified with a 10% EDTA (pH 7.4, Ling Feng Chemical Reagent Co., LTD, Shanghai, China) decalcifying solution at room temperature for 4 weeks. After decalcification, the glass ionomer cement and resin composite was carefully removed from the cavity and rinsed with phosphate-buffered saline (PBS, pH 7.4) three times. The specimens were then dehydrated in ascending grades of ethanol, dealcoholized by xylene, and embedded in paraffin. Serial sections of 5 μm thickness were cut using a sliding microtome (Leica Microsystems, Vertrieb, Wetzlar, Germany) for histopathological and immunohistochemical examination [21].

### Specimen staining

Hematoxylin-Eosin staining was used for observing the response of pulp tissue to the pulp capping materials. ICI, PTD and RDF were evaluated according to the criteria of modified versions of the ISO 10993 and 7405 standards presented in Tables 1–3 [22]. The performances of the two materials were compared, and the results were recorded[23].

**Table 1:** Inflammatory Cell Infiltration (ICI).

Inflammatory cell infiltration	Inflammatory cell infiltration Characterization
Score 0	None or a few scattered inflammatory cells present in the pulp area corresponding to the pulp exposure
Score 1	Slight inflammatory cell infiltrate with polymorphonuclear (PMNs) or mononuclear leukocytes (MNLs)
Score 2	Moderate inflammatory cell infiltrate involving the coronal pulp
Score 3	Severe inflammatory cell infiltrate involving the coronal pulp or characterizing abscess

**Table 2:** Pulp Tissue Disorganization (PTD).

Tissue disorganization	Characterization
Score 0	Normal tissue
Score 1	Odontoblastic layer disorganization but central pulp normal
Score 2	Total disorganization of the pulp tissue morphology
Score 3	Pulp necrosis

**Table 3:** Reparative Dentin Formation (RDF).

Reactionary or reparative dentin formation	Characterization
Score 0	Absence
Score 1	Modest hard tissue deposition beneath the exposed area
Score 2	Moderate hard tissue deposition beneath the exposed area
Score 3	Intense hard tissue deposition beneath the exposed area characterizing complete dentin bridge

Immunohistochemical staining was used for observing the expression of Runx2, COL I, OCN, and DSP, which were expressed primarily at the early stage of reparative dentinogenesis. The sections were deparaffinized with xylene, hydrated in a series of descending grades of ethanol, and then rinsed briefly with PBS for the primary antibodies; the sections were incubated overnight at 4°C with polyclonal anti- Runx2, COL I, OCN and DSP (Wuhan Boster Biological Technology, Wuhan, China). They were then rinsed with PBS three times for 5 min each and immunochemically stained using StreptAvidin Biotin Complex (SABC) methods. The antibody localized antigen was then detected by peroxidase activation of 3, 30-diaminobenzidine (DAB, Wuhan Boster Biological Technology, Wuhan, China.) for 5 min. Finally, the sections were counterstained with Mayer’s hematoxylin. The stained sections with root canal orifice fully shown were observed under a light microscope(Leica DM 4000B, German), the images were analyzed with Image Pro Plus 6.0 software (MediaCybernetics, USA) for quantification of the integrated optical density (IOD) and the corresponding area. The mean optical density was used for statistical analysis.

### Statistical analysis

The results of histopathological evaluation were statistically analyzed by the Mann-Whitney U-test, testing for differences between each experimental group and the positive control, as well as the blank control, during each observation period at a significance level of 0.05.

Immunohistochemical evaluation data were expressed as the mean ±standard error, results were analyzed using ANOVA and Student *t* test, P < 0.05 was considered significant different using statistical software (SPSS 17.0 Base System SC, IBM SPSS, China).

## Results

### Histological observation

**Fig 1:**
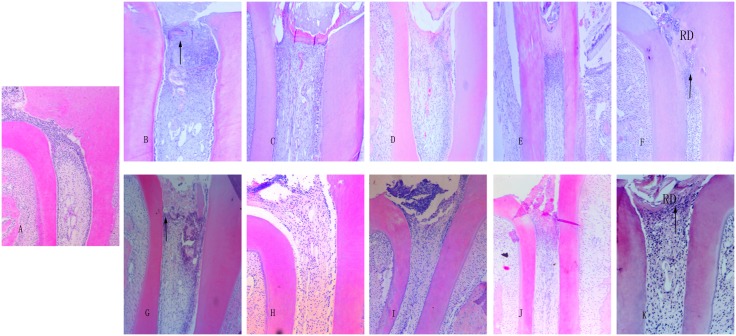
Representative histological images of the Ca(OH)_2_ and DBM groups (x100 magnification, H&E staining). A: The blank control group. Pulp morphology was normal. B-F: Ca(OH)_2_ group (1, 3, 7, 14, and 28 days). G-K: DBM group (1, 3, 7, 14, and 28 days). Inflammatory reactions began from 1 day and continued to exist through the observation period. Reparative dentin formation could be observed in both groups on 28 days, and the DBM group showed more regular and thinner reparative dentin. Arrowheads indicate inflammatory reaction. RD means reparative dentin.

**Fig 2:**
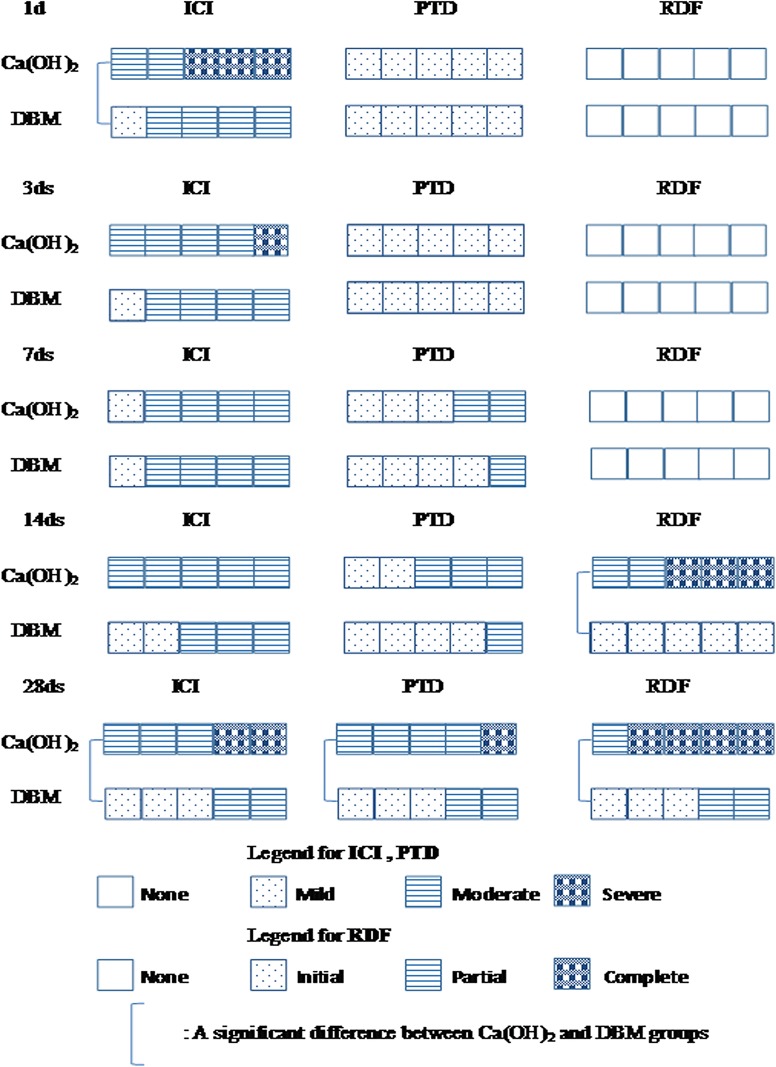
Results of the histopathological evaluation. ICI: Inflammatory cell infiltration, PTD: Pulp tissue disorganization, RDF: Reparative dentin formation.

Representative histopathological images of the groups were shown in Fig 1. A summary of the results of the histopathological evaluation was given in Fig 2. Normal pulp morphology as the baseline could be observed in the blank control group. On 1 day, a substantial amount of inflammatory cell infiltration could be detected in both Ca(OH)_2_ and DBM groups and was more obvious in Ca(OH)2 group with the coagulation necrosis beneath. On 3 days and 7 days, the inflammatory reaction decreased gradually, matrix secretion could be observed in both groups, and this response increased in a time-dependent manner. On 14 days, the matrix calcification could be observed, and it was more obvious in the Ca(OH)_2_ group. On 28 days, inflammatory cell infiltration could still be found. In the DBM group, the structure of the pulp tissue beneath was basically normal, while in the Ca(OH)_2_ group, pathological calcification or necrosis of the pulp tissue was observed. Dentin bridges could be observed in both groups.

Both the Ca(OH)_2_ and DBM groups showed mild-to-moderate level ICI and PTD at all observation sites. The results of the Mann-Whitney U-test for the histopathological evaluation showed slighter ICI on 1 and 28 days and milder PTD on 28 days in DBM groups compared with the Ca(OH)_2_ group with significant difference (P<0.05). Reparative dentin formation (RDF) could initially be observed on 14 days postoperatively in the two groups. DBM group showed less RDF with significant difference on 14 and 28 days compared with the Ca(OH)_2_ group (P<0.05).

### Immunohistochemical observation

**Fig 3:**
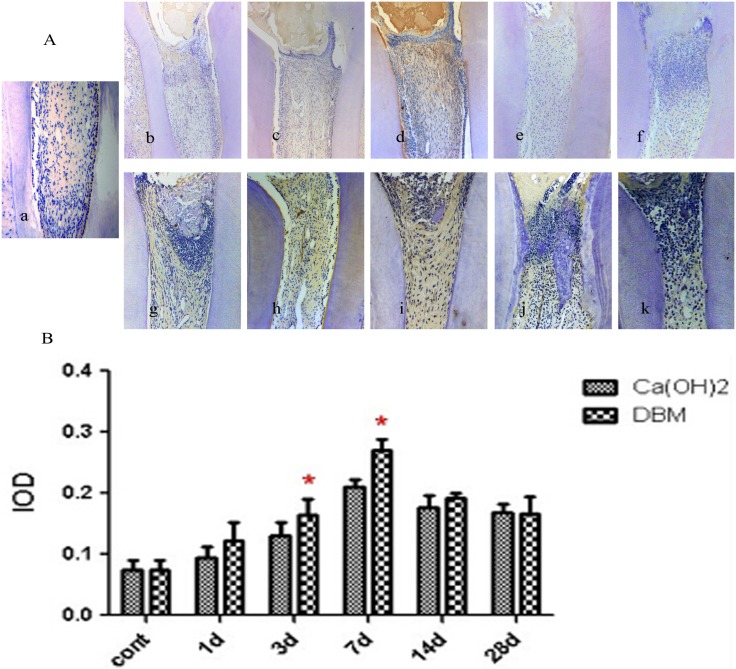
Immunohistochemical expression of Runx2. (A): Representative immunohistochemical images of the blank control group, the Ca(OH)_2_ group and the DBM group. a: The blank control group. Pulp morphology was normal. b- f: Ca(OH)_2_ group (1, 3, 7, 14, 28 days). g- k: DBM group (1, 3, 7, 14, 28 days). (B): Mean IOD value of Runx2. *means significant difference.

**Fig 4:**
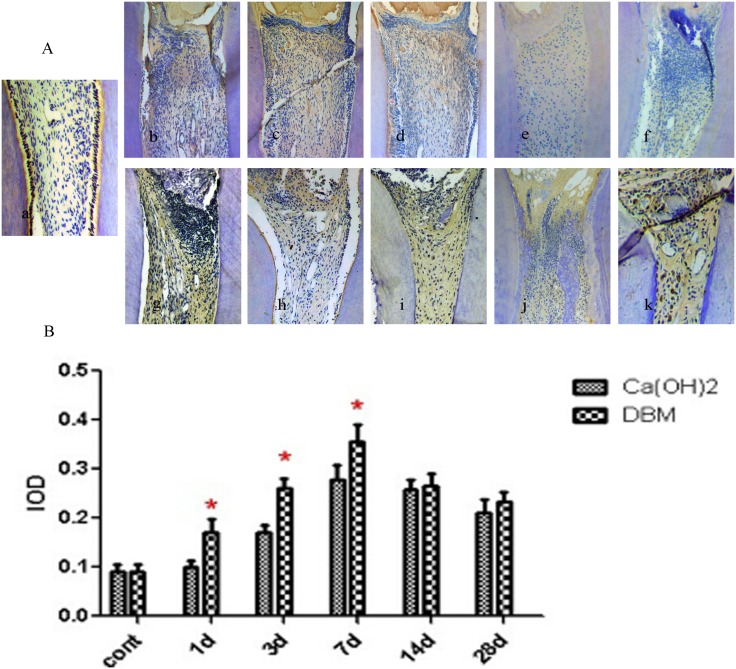
Immunohistochemical expression of COL I. (A): Representative immunohistochemical images of the blank control group, the Ca(OH)_2_ group, and the DBM group. a: The blank control group. Pulp morphology was normal. b- f: Ca(OH)_2_ group (1, 3, 7, 14, 28 days). g- k: DBM group (1, 3, 7, 14, 28 days). (B): Mean IOD value of COL I. *means significant differences.

The early mineralization-related factors selected to study as specific markers of dentinogenesis were Runx2 and COL I. Positive expression of Runx2 and COL I were mainly in the preodontoblasts. The expression trend of the two factors in the Ca(OH)_2_ and DBM groups was consistent. It increased gradually from 1 day and reached a peak in approximately 7 days. Then, it decreased on 14 and 28 days. Generally, in the observation periods from 1 day to 28 days, Runx2 and COL-1 expression in the DBM group were higher than the Ca(OH)_2_ group; the difference was statistically significant on 3 and 7 days for Runx2, and on 1, 3, and 7 days for COL-1 (p <0.05) (Figs 3 and 4).

**Fig 5:**
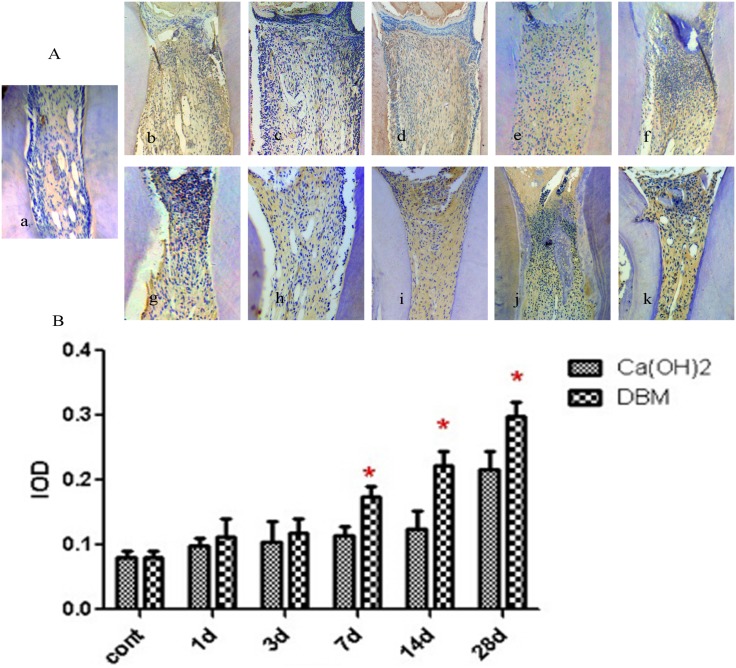
Immunohistochemical expression of OCN. (A): Representative immunohistochemical images of the blank control group, the Ca(OH)_2_ group, and the DBM group. a: The blank control group. Pulp morphology was normal. b- f: Ca(OH)_2_ group (1, 3, 7, 14, 28 days). g- k: DBM group (1, 3, 7, 14, 28 days). (B): Mean IOD value of OCN. *means significant differences.

**Fig 6:**
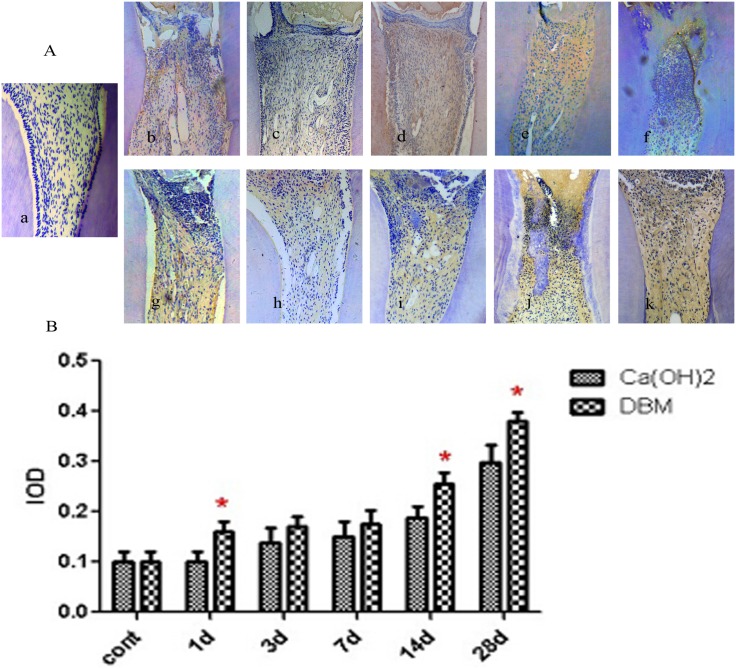
Immunohistochemical expression of DSP. (A): Representative immunohistochemical images of the blank control group, the Ca(OH)_2_ group, and the DBM group. a: The blank control group. Pulp morphology was normal. b- f: Ca(OH)_2_ group (1, 3, 7, 14, 28 days). g- k: DBM group (1, 3, 7, 14, 28 days). (B): Mean IOD value of DSP. *means significant differences.

The late mineralization-related factor OCN and specific odontoblastic marker DSP were mainly positively expressed in predentin and odontoblasts. The expression trend of OCN and DSP in the two groups was consistent. The trend enhanced gradually from 1 to 28 days. Generally, from 1 to 28 days, their expression in the DBM group was higher than the Ca(OH)_2_ group. The difference was statistically significant on 7, 14, and 28 days for OCN and on 1, 14, and 28 days for DSP (p <0.05) (Figs 5 and 6).

## Discussion

In our study, pulp capped with DBM had slighter inflammation reaction and a more obvious mineralization compared with Ca(OH)_2_ group. This suggested that as a biological material, DBM had appropriate biocompatibility and ability for physiological pulp repair. From the histopathological observation, many cells could be found in DBM layers. The pore size of DBM, ranging from 350 to 750 μm, was matched with the cell size and could thus induce the invasion of new blood vessels within its 3D structure [24]. Therefore, the cell- and vessel-rich spongy structure may play the role of a cushion to relieve inflammatory exudates, the histological elasticity was also improved to some extent.

Ideal pulp capping material should not only provide suitable microenvironment for dentin biomineralization but also should have certain antibacterial abilities, helping resist further injury aroused by the residual bacteria in dental pulp tissue. A steady and healthy pulp healing environment is of essential significance to pulp repair. In our study, it could be found that inflammatory cells infiltrated into whole dental pulp tissue in several Ca(OH)_2_ samples, resulting in dental pulp necrosis, even abscess formation. The alkaline pH of Ca(OH)_2_ may be an irritant to pulp tissue, making the pulp inflamed beyond control. Compared with the Ca(OH)_2_ group, DBM showed limited inflammation response beneath the pulp capping agent; the existence of inflammation suggests that DBM has no antibacterial role. Ca(OH)_2_ provides a mass of calcium, leading to calcium phosphate deposition and amorphous calcified mass formation [25]. The DBM group had thinner but more regular dentin bridge formation. Clinically, the use of Ca(OH)_2_ in pulpotomy of teeth frequently results in thick calcified bridge formation, making the root canal orifice blocked. Therefore, the formation of reparative dentin bridges with proper thicknesses to protect pulp vitality meanwhile reducing clinical dilemma is essential for pulp capping materials.

We selected the four related biological active factors that express the physiological dentinogenesis stage to investigate the reparative dentin formation progress. The odontoblast differentiation pattern is similar to osteoblast in gene and protein expression levels. Runx2, as a specific transcription factor, plays an important role in transcription regulation of odontoblasts differentiation [26–28]. COL I accounts for a large proportion in the predentin and dentin matrix, and it is one of the markers of early odontoblast differentiation [29]. OCN expresses in the late osteoblast differentiation mature stage, so we consider OCN an index of relative specificity markers of late odontoblasts differentiation [30]. DSP is a non-collagen protein, and it is essential in dentin mineralization. The phosphorus proteins nucleate to initiate dentin mineralization and crystal growth [31]. It was found that the DBM group showed a higher expression on those four factors, which may be associates with the BMPs. The role BMP2 plays in dental pulp stem cell differentiation, through BMP2—Smad1/5—DPC4—RUNX2 pathways, was already verified [32]. Runx2 gene binding sites are widespread in COL I, ALP, and DSP relevant genes, indicating that BMP2 plays an important role in odontoblast differentiation. Runx2, as a transcription factor, can also promote the expression of osteoblast-specific genes such as OCN and COL I [33]. The non-collagen proteins secreted by the odontoblast are gathered to the dentin matrix, promoting and regulating the mineralization of dentin matrix.

There are many biologically active factors participating in dental pulp stem cell proliferation and differentiation. Although DBM contains BMPs, the content is relatively low, and its release may be discontinuous, which may lead to discontinuous formation of the dentin bridge. If growth factors such as VEGF and BMP2 were added to DBM, better clinical effects may be obtained, this subject should be studied further.

On the basis of the findings from this study, the null hypothesis that DBM comprised of BMPs and COL I would promote wound healing and dentin bridge formation on the exposed rat pulp was accepted. However, DBM itself has no anti-inflammatory effect, so in subsequent trials, it may be considered appropriately to add a dosage of antibacterial drugs to obtain a better dental pulp capping effect.

## References

[1] Baume LJ, Holz J. Long term clinical assessment of direct pulp capping. International dental journal. 1981;31(4):251–60. 7030965

[2] Raedel M, Hartmann A, Bohm S, Konstantinidis I, Priess HW, Walter MH. Outcomes of direct pulp capping: interrogating an insurance database. International endodontic journal. 2015. 10.1111/iej.12564 26474914

[3] Swarup SJ, Rao A, Boaz K, Srikant N, Shenoy R. Pulpal response to nano hydroxyapatite, mineral trioxide aggregate and calcium hydroxide when used as a direct pulp capping agent: an in vivo study. J Clin Pediatr Dent. 2014;38(3):201–6. 25095313 10.17796/jcpd.38.3.83121661121g6773

[4] Hilton TJ. Keys to clinical success with pulp capping: a review of the literature. Oper Dent. 2009;34(5):615–25. 10.2341/09-132-0 19830978 PMC2856472

[5] Foreman PC, Barnes IE. Review of calcium hydroxide. International endodontic journal. 1990;23(6):283–97. 2098345 10.1111/j.1365-2591.1990.tb00108.x

[6] Parirokh M, Torabinejad M. Mineral trioxide aggregate: a comprehensive literature review--Part III: Clinical applications, drawbacks, and mechanism of action. J Endod. 2010;36(3):400–13. 10.1016/j.joen.2009.09.009 20171353

[7] Briso AL, Rahal V, Mestrener SR, Dezan E Junior. Biological response of pulps submitted to different capping materials. Braz Oral Res. 2006;20(3):219–25. 17119704 10.1590/s1806-83242006000300007

[8] Islam I, Chng HK, Yap AU. Comparison of the physical and mechanical properties of MTA and portland cement. J Endod. 2006;32(3):193–7. 10.1016/j.joen.2005.10.043 16500224

[9] Iwata H, Sakano S, Itoh T, Bauer TW. Demineralized bone matrix and native bone morphogenetic protein in orthopaedic surgery. Clin Orthop Relat Res. 2002(395):99–109. 11937869 10.1097/00003086-200202000-00010

[10] Pabaney AH, Reinard KA, Asmaro K, Malik GM. Novel technique for cranial reconstruction following retrosigmoid craniectomy using demineralized bone matrix. Clin Neurol Neurosurg. 2015;136:66–70. 10.1016/j.clineuro.2015.05.034 26067724

[11] Rabie AB. Vascular endothelial growth pattern during demineralized bone matrix induced osteogenesis. Connect Tissue Res. 1997;36(4):337–45. 9610891 10.3109/03008209709160232

[12] Zimmermann G, Moghaddam A. Allograft bone matrix versus synthetic bone graft substitutes. Injury. 2011;42 Suppl 2:S16–21. 21889142 10.1016/j.injury.2011.06.199

[13] Bouvier M, Joffre A, Magloire H. In vitro mineralization of a three-dimensional collagen matrix by human dental pulp cells in the presence of chondroitin sulphate. Arch Oral Biol. 1990;35(4):301–9. 2116121 10.1016/0003-9969(90)90047-e

[14] Saito T, Ogawa M, Hata Y, Bessho K. Acceleration effect of human recombinant bone morphogenetic protein-2 on differentiation of human pulp cells into odontoblasts. J Endod. 2004;30(4):205–8. 10.1097/00004770-200404000-00005 15085046

[15] Liu X, Li Y, Zhao B. [Experimental study on effect of capping pulp with collagen]. Zhonghua Kou Qiang Yi Xue Za Zhi. 2001;36(6):448–50. 11930723

[16] Pampaloni F, Reynaud EG, Stelzer EH. The third dimension bridges the gap between cell culture and live tissue. Nat Rev Mol Cell Biol. 2007;8(10):839–45. 10.1038/nrm2236 17684528

[17] Bhatia M. Hematopoiesis from human embryonic stem cells. Ann N Y Acad Sci. 2007;1106:219–22. 10.1196/annals.1392.007 17332088

[18] Yang X, van der Kraan PM, Bian Z, Fan M, Walboomers XF, Jansen JA. Mineralized tissue formation by BMP2-transfected pulp stem cells. J Dent Res. 2009;88(11):1020–5. 10.1177/0022034509346258 19828890

[19] Chen B, Lin H, Zhao Y, Wang B, Zhao Y, Liu Y, et al Activation of demineralized bone matrix by genetically engineered human bone morphogenetic protein-2 with a collagen binding domain derived from von Willebrand factor propolypeptide. Journal of biomedical materials research Part A. 2007;80(2):428–34. 10.1002/jbm.a.30900 17013862

[20] Zhang M, Fukuyama H. CGRP immunohistochemistry in wound healing and dentin bridge formation following rat molar pulpotomy. Histochem Cell Biol. 1999;112(5):325–33. 10603071 10.1007/s004180050413

[21] Fransson H, Petersson K, Davies JR. Dentine sialoprotein and collagen I expression after experimental pulp capping in humans using emdogain gel. International endodontic journal. 2011;44(3):259–67. 10.1111/j.1365-2591.2010.01824.x 21166828

[22] de Souza Costa CA, Lopes do Nascimento AB, Teixeira HM, Fontana UF. Response of human pulps capped with a self-etching adhesive system. Dent Mater. 2001;17(3):230–40. 11257296 10.1016/s0109-5641(00)00076-2

[23] Suzuki M, Taira Y, Kato C, Shinkai K, Katoh Y. Histological evaluation of direct pulp capping of rat pulp with experimentally developed low-viscosity adhesives containing reparative dentin-promoting agents. J Dent. 2016;44:27–36. 10.1016/j.jdent.2015.11.005 26620099

[24] Mondalek FG, Lawrence BJ, Kropp BP, Grady BP, Fung KM, Madihally SV, et al The incorporation of poly(lactic-co-glycolic) acid nanoparticles into porcine small intestinal submucosa biomaterials. Biomaterials. 2008;29(9):1159–66. 10.1016/j.biomaterials.2007.11.020 18076986 PMC2947939

[25] Ito S, Saito T, Amano K. In vitro apatite induction by osteopontin: interfacial energy for hydroxyapatite nucleation on osteopontin. Journal of biomedical materials research Part A. 2004;69(1):11–6. 10.1002/jbm.a.20066 14999746

[26] Camilleri S, McDonald F. Runx2 and dental development. Eur J Oral Sci. 2006;114(5):361–73. 10.1111/j.1600-0722.2006.00399.x 17026500

[27] Torneck CD, Moe H, Howley TP. The effect of calcium hydroxide on porcine pulp fibroblasts in vitro. J Endod. 1983;9(4):131–6. 10.1016/S0099-2399(83)80031-4 6574198

[28] Han N, Zheng Y, Li R, Li X, Zhou M, Niu Y, et al beta-catenin enhances odontoblastic differentiation of dental pulp cells through activation of Runx2. PloS one. 2014;9(2):e88890 10.1371/journal.pone.0088890 24520423 PMC3919828

[29] Gaikwad JS, Hoffmann M, Cavender A, Bronckers AL, D'Souza RN. Molecular insights into the lineage-specific determination of odontoblasts: the role of Cbfa1. Adv Dent Res. 2001;15:19–24. 10.1177/08959374010150010501 12640733

[30] Owen TA, Bortell R, Yocum SA, Smock SL, Zhang M, Abate C, et al Coordinate occupancy of AP-1 sites in the vitamin D-responsive and CCAAT box elements by Fos-Jun in the osteocalcin gene: model for phenotype suppression of transcription. Proc Natl Acad Sci U S A. 1990;87(24):9990–4. 2124710 10.1073/pnas.87.24.9990 PMC55300

[31] Yamamoto R, Oida S, Yamakoshi Y. Dentin Sialophosphoprotein-derived Proteins in the Dental Pulp. J Dent Res. 2015;94(8):1120–7. 10.1177/0022034515585715 25951824

[32] Chen PY, Sun JS, Tsuang YH, Chen MH, Weng PW, Lin FH. Simvastatin promotes osteoblast viability and differentiation via Ras/Smad/Erk/BMP-2 signaling pathway. Nutr Res. 2010;30(3):191–9. 10.1016/j.nutres.2010.03.004 20417880

[33] Jeon EJ, Lee KY, Choi NS, Lee MH, Kim HN, Jin YH, et al Bone morphogenetic protein-2 stimulates Runx2 acetylation. The Journal of biological chemistry. 2006;281(24):16502–11. 10.1074/jbc.M512494200 16613856

